# Traditional infant oil massage in early life: a cross-sectional study of knowledge and practices among young mothers in Malappuram district, Kerala

**DOI:** 10.3389/fmed.2026.1779228

**Published:** 2026-05-08

**Authors:** Keerthi R. Panicker, Anusree Dileep, S. Meera, M. Vandana Rani, Pillai Deepthi Pradeepan, Delvin T. Robin

**Affiliations:** Department of Swasthavritta (Social and Preventive Medicine), Amrita School of Ayurveda, Amritapuri, Amrita Vishwa Vidyapeetham, Kollam, India

**Keywords:** abhyaṅga, Āyurveda, dinacaryā, paediatric oil massage, svastha

## Abstract

**Background and objectives:**

Āyurveda recommends abhyaṅga (massage) for swastha (healthy individual) in the context of jātamātra paricharya (neonatal care) and in Ayurvedic treatises such as Arogyakalpadrumam as a daily routine. There have been many community surveys held in India to explore the prevalence and various practices of infant oil massage tradition, but there is a void in data regarding the current status of traditional infant oil massage practice among communities in the Kerala state. Hence, this study was conducted to understand the knowledge level and current practices related to traditional infant oil massage.

**Method:**

A cross-sectional survey was conducted among 145 primiparous mothers of infants in Malappuram district, Kerala. The data were collected via face-to-face interview on their consent using a newly developed closed-ended questionnaire after testing its validity and reliability.

**Results:**

In total, 71% of mothers were practicing traditional oil massage to their infants, 94.5% of study subjects were unaware about abhyaṅga said in Āyurveda, and 96.9% never sought opinion from Ayurvedic practitioner before starting massage practice. Coconut oil was used by 75.9% mothers.

**Conclusion:**

Traditional infant oil massage practices were widely practiced across the study setting. The knowledge level of mothers related to this traditional practice seemed limited, and the practices were varied too. Therefore, authentic, scientific, and systematic guidance from reliable sources to parents and caretakers may serve as a better infant healthcare approach. Thus, Āyurveda may help in shaping policies and protocols to sculpt a healthy life by incorporating such infant care modalities.

## 1 Introduction

Pertinent and realistic public health measures mandate a widespread embrace of economical and evidence-based healthcare initiatives for the care of infant health. Studies on infant care practices establish the high prevalence of infant oil massage in various Asian nations (1–3) and states of India such as Maharashtra, Madhya Pradesh (4), and Karnataka (5) indicating the robust and extensive cultural acceptance of this practice in different societies. Gentle oil massage with non-irritant oil is included in the protocol for the care of normal neonates as published in the AIIMS Protocols in Neonatology (2019) (6). The Indian Academy of Pediatrics endorses oil massage with level II evidence in their evidence-based guidelines for pediatric skin care (7). Infant oil massage is perceived to have benefits such as thermoregulation, improved weight gain, circulation, and quality of sleep along with local effects on the skin (8).

Āyurveda recommends the healthy individuals to practice abhyaṅga everyday as part of dinacaryā in the context of jātamātra paricharya (9). Pediatric massage is mentioned. Ārogyakalpadrumam, the vernacular Āyurvedic pediatric text, quotes the use of coconut milk for daily massage in healthy children (10). Despite a well-established tradition in Kerala, there are limited data on the prevalence and practices of traditional infant oil massage in the state. In addition, there is a lack of state-wide institutional training or expert guidance given to the mother or caregiver regarding the practice of traditional infant oil massage. Malappuram district was selected for the present study based on the Annual Vital Statistics Report (2019), which identifies it as having the highest crude birth rate among the 14 districts of Kerala, thereby providing a larger and relevant population for studying infant care practices (11). To ensure homogeneity of the study population and to minimize bias arising from prior experience in infant care practices, primiparous mothers were specifically selected for this study. This approach enables a more accurate assessment of baseline knowledge, attitudes, and practices related to infant oil massage.

Hence, a cross-sectional survey was conducted among 145 young primiparous mothers of infants belonging to five randomly selected health blocks in Malappuram district. The result is expected to serve as the potential baseline data that can be made use of in health education as well as better healthcare practices if given the appropriate scientific attention and understanding thus augmenting the science of Āyurveda.

## 2 Objectives

The objectives of this study are as follows:

- To assess the knowledge level regarding infant oil massage tradition among the young mothers in Malappuram district, Kerala.
- To identify the current practices related to the traditional infant oil massage among the young mothers in Malappuram district, Kerala.

## 3 Methods

### 3.1 Study design

The study employed a cross-sectional survey approach.

### 3.2 Method of data collection

A closed-ended questionnaire with socio-demographic details and 26 items was developed for collecting the data. Face validity, content validity, construct validity (convergent and discriminant), and reliability (internal consistency) of the tool were determined. The final valid and reliable questionnaire with 25 items and five domains namely status of infant oil massage practice, knowledge, attitude, practice, and selection and usage of oil was used for the survey.

### 3.3 Sample characteristics

A total of 145 young primiparous mothers of the infant were selected using a two-stage random sampling method from five randomly selected (lottery method) health blocks—Maranchery, Thavanur, Kuttippuram, Vettom, and Vengara—in Malappuram District. In the second stage, the participants were randomly selected from maternal and child health (MCH) registries maintained at the respective centers, which have near-universal coverage due to Kerala’s robust primary healthcare system and active postnatal follow-up. Thus, the sampling frame included registered beneficiaries from the community and was not limited to walk-in facility attendees, enhancing the representativeness of the study population.

#### 3.3.1 Inclusion criteria

The inclusion criteria were as follows:

- Primiparous mothers of infants (infant’s age >1 week and less than or equal to 12 months).
- Mother’s age—21 to 29 years.
- Mothers with infants born on term.

#### 3.3.2 Exclusion criteria

The exclusion criteria were as follows:

- Subjects who are not willing to participate in the study.
- Mother of pre-term baby (<37 completed weeks of gestation).
- Mother of post-term baby (born beyond 2 weeks of the EDD, i.e., >294 days).
- Mothers of low-birth-weight babies (<2.5 kg).
- Mothers whose infants are diagnosed with any systematic disease, congenital disorders, or deformities.

### 3.4 Sample size

The sample size was calculated using GPower Version 3.1.9 software using the formula given below.


$$N=(({\left(Z_{1-\alpha /2}+Z_{1-\beta }\right)}^{2}))/d^{2}$$

where *N* is the sample size required, *d* (effect size) = 0.3, *α* = level of significance = 0.05, *β* = 1 − Power = 0.05, *Z*(1 − *α*/2) = 1.96, and *Z*(1 − *β*) = 1.645.

#### 3.4.1 Calculation

The calculation was calculated using the formula given below.


$$N=({\left(1.96+1.645\right)}^{2})/(0.3^{2})=144.4\approx 145$$

### 3.5 Survey administration

Face-to-face survey was administered at the head institutions (Community Health Centers/Taluk hospital) of each of the selected health blocks in Malappuram district. The total duration was 18 months.

### 3.6 Ethical consideration

Survey began after obtaining IEC approval and consent from the participants. The collected data were de-identified by providing a code and stored in excel sheet which was password protected to prevent unauthorized access (Ethics approval number—IEC-AIMS-2022-AYUR-057).

### 3.7 Composite score development and statistical analysis

To quantitatively assess the level of knowledge and practice related to traditional infant oil massage among the study participants, composite scores were developed using selected items from a validated questionnaire. The statistical objective was to assess the association between knowledge and practice scores among study participants using correlation analysis.

#### 3.7.1 Knowledge domain score

The knowledge score was derived from items Q5, Q6, Q7, Q9, and Q11, selected to capture key cognitive components of traditional infant oil massage, namely, awareness of authentic Ayurvedic references (Q5), knowledge of contraindications (Q6), perceived benefits and understanding of the practice (Q7), consultation with qualified Ayurvedic practitioners (Q9), and rationale behind oil selection (Q11). These items collectively reflect awareness, conceptual understanding, and informed decision-making central to the knowledge construct. Each item was scored dichotomously (correct/aware = 1; incorrect/unaware = 0) and summed to yield a total score ranging from 0 to 5, with higher scores indicating better knowledge. Practice items were scored on an ordinal scale (0–3), where 0 = not aligned, 1 = partially aligned, 2 = moderately aligned, and 3 = fully aligned with recommended/traditional practices; the summed practice score ranged from 0 to 24. The criteria for “recommended practice” were based on classical Ayurvedic textual references (e.g., dinacaryā and jātamātra paricharyā), standard pediatric guidelines from the Indian Academy of Pediatrics, and expert consensus during tool validation (content validation by five subject experts). As the composite scores were derived from ordinal and non-normally distributed data, median and interquartile range (IQR) were used to describe distribution (knowledge: median = 2, IQR = 2; practice: median = 11, IQR = 3). Missing values were minimal and handled by excluding incomplete responses from domain-specific score calculations. Items were combined into composite scores to represent overall knowledge and practice constructs, with all items equally weighted, as they reflect related dimensions of the same domain.

#### 3.7.2 Practice domain score

The practice domain assessed the implementation and quality of infant oil massage, including initiation, continuation, motivation, timing, frequency, duration, technique, type of oil used, and preparatory procedures. Practice scores were computed from items Q1, Q8, Q10, Q12, Q14, Q16, Q18, and Q19, which collectively capture behavioral execution, consistency, and procedural appropriateness of the practice. Specifically, these items addressed initiation and continuation (Q1, Q8), motivation and intention (Q10), timing of commencement (Q12), frequency and duration (Q14), techniques and procedural aspects (Q16), type and source of oil (Q18), and preparatory practices such as oil heating (Q19). Responses were scored on an ordinal scale (0–3), where 0 = not aligned, 1 = partially aligned, 2 = moderately aligned, and 3 = fully aligned with recommended or traditional standards. The criteria for recommended practice were based on classical Ayurvedic textual references (e.g., dinacaryā and jātamātra paricharyā), standard pediatric guidelines from the Indian Academy of Pediatrics, and expert consensus during tool validation. The summed practice score reflected overall adherence, with higher scores indicating better practice levels.

#### 3.7.3 Statistical analysis

SPSS and Smart PLS-SEM software were used for statistical analyses. Structural equation modeling of the pilot data was performed. Frequency percentage, charts, and graphs were used to present survey results. Spearman’s rank-order correlation was done to assess the relationship between knowledge and practice domain.

## 4 Results

### 4.1 Socio-demographic status

The study surveyed 145 primiparous mothers in Malappuram district, Kerala, providing key insights into their demographic profiles, knowledge, attitude, and practice regarding the traditional infant oil massage. The study participants were in the age group 21 to 29 years; of that majority was under the age of 25 years (90.9%). The majority were Muslims (70.3%, *n* = 102) followed by Hindu (29.7%, *n* = 43). Predominantly, the occupation of the mothers was housewife (93.1%, *n* = 135). The age of the infants under study were observed as 14.48% under 3 months, 34.48% between 3 and 6 months, 25.52% between 6 and 9 months, and 25.52% between 9 and 12 months. Gender distribution of the infants shows that 51% (*n* = 74) were girls and 49% (*n* = 71) were boys (Table 1).

**Table 1:** Socio-demographic characteristics of young mothers and infants in Malappuram district participating in the study.

Variables	Category	Count	Percentage
Age of mother	21–25	132	90.9
	26–29	13	9.1
Distribution of religion	Hindu	43	29.7
	Muslim	102	70.3
Marital status	Married	145	100
Education status of mothers	10th	2	1.4
	12th	44	30.3
	University	99	68.3
Occupation of mothers	Housewife	135	93.1
	Private sector	6	4.1
	Self-employed	4	2.8
Socio-economic status	High	17	11.7
	Middle	95	65.5
	Poor	33	22.8
Gender of child	Boy	71	49.0
	Girl	74	51.0
Age of infant	Up to 3 months	21	14.48
	3–6 months	50	34.48
	6–9 months	37	25.52
	9–12 months	37	25.52
Mode of delivery	Abdominal	31	21.4
	Vaginal	114	78.6
Place of delivery	Government hospital	7	4.8
	Government medical college	1	0.7
	Private hospital	137	94.5

### 4.2 Status of infant oil massage practice

Out of the 29% mothers who were not practicing infant oil massage, 18.6% replied that they had once practiced but discontinued it later and the remaining 10.3% had never given traditional oil massage to their infants. The reasons for never practicing massage were reported as unavailability of skilled person (1.4%), mother’s own choice (3.4%), or advised by registered medical professional (5.5%). The reason for discontinuance of massage practice by 18.6% mothers out of 145 were reported as unavailability of skilled person (2.1%), mother’s own choice (4.8%), or advised by registered medical professional (11.7%) (Table 2).

**Table 2:** Status of infant oil massage practices among young mothers in Malappuram district (*n* = 145).

Variable	Category	*n*	% (overall)
Current practice of infant oil massage	Yes	103	71.0
	No	42	29.0
Among mothers who never practiced infant oil massage (*n* = 15)			
Reason for never practicing	Advised by registered medical professional	8	53.3^*^
	No skilled person available	2	13.3^*^
	Own choice/no specific reason	5	33.3^*^
Among mothers who discontinued infant oil massage (*n* = 27)			
Reason for discontinuation	Advised by doctor due to illness	17	63.0^*^
	No skilled person available	3	11.1^*^
	Own choice	7	25.9^*^

Values are presented as frequency (*n*) and percentage (%). Percentages marked with an asterisk (*) are calculated within subgroup denominators (never practiced, *n* = 15; discontinued, *n* = 27). Overall percentages are based on the total sample (*n* = 145).

### 4.3 Knowledge of infant oil massage practice

In total, 94.5% of mothers did not know about the authentic reference for oil massage in Āyurveda, but 5.5% knew the same. Majority of mothers (77.9%) knew that there are conditions where oil massage is contra indicated. A total of 12.4% of mothers did not know about the same, and 9.7% said that they were uncertain about it. Most of the mothers (73.1%) opined that traditional infant oil massage practice was beneficial, 22.1% replied that they had no opinion or preferred not to answer, and 4.8% responded as non-beneficial (Table 3).

**Table 3:** Knowledge levels related to infant oil massage practice among young mothers (*n* = 145).

Variable	Category	*n*	%
Awareness of authentic Ayurvedic reference for oil massage	Yes	8	5.5
	No	137	94.5
Awareness of conditions where massage should be avoided	Yes	113	77.9
	No	18	12.4
	Uncertain/do not know	14	9.7
Opinion regarding traditional infant oil massage	Beneficial	106	73.1
	Not beneficial	7	4.8
	No opinion/do not know	32	22.1

Values are expressed as frequency (*n*) and percentage (%), calculated based on the total sample size (*n* = 145).

### 4.4 Mothers’ attitude toward traditional infant oil massage practice

The reason for starting traditional infant oil massage practice was reported as a traditional practice by all 130 mothers. The remaining 15 had never given the massage. The data show that 86.9% of mothers never sought the opinion of any registered Āyurvedic practitioner before starting massage practice. Only 2.8% of mothers did seek the opinion of any registered Āyurvedic practitioner before starting massage practice. Table 4 shows that 60% were planning to continue/restart this practice of oil massage to their children whereas 13.8% mothers were planning not to continue/restart this practice. Approximately 15.9% were uncertain about the same. The data say that 40% of mothers recommend infant oil massage to others, 11.7% chose not to recommend it, and 37.9% had no opinion (Table 4).

**Table 4:** Attitudes of young mothers toward infant oil massage practice.

Variable	Category	*n*	%
Among mothers who practiced infant oil massage (*n* = 130)			
Reason for initiating massage	Traditional practice	130	100.0^*^
Sought opinion of registered Ayurvedic practitioner before starting	Yes	4	3.1^*^
	No	126	96.9^*^
Among all study participants (*n* = 145)			
Planning to continue/restart practice	Yes	87	60.0
	No	20	13.8
	Uncertain	23	15.9
Recommendation of infant oil massage to others	Yes	58	40.0
	No	17	11.7
	No opinion	55	37.9

Values are presented as frequency (*n*) and percentage (%). Percentages marked with an asterisk (*) are calculated using the subgroup denominator (mothers who practiced infant oil massage, *n* = 130). Remaining percentages are calculated using the total sample size (*n* = 145).

### 4.5 Selection and usage of oil

Virgin/normal coconut oil was found to be the majority’s (75.9%) choice for head massage. In total, 12.4% were using medicated Āyurvedic oil, and 1.4% were using any baby oil manufactured by leading pharmaceutical companies for massaging head. The majority (67.6%) chose virgin/normal coconut oil for body massage. 16.6% were using medicated Āyurvedic oil, whereas 3.4% were using any baby oil manufactured by leading pharmaceutical companies for body massage. The choice of oil was purely based on their traditional knowledge as reported by 82.8% of mothers. Approximately 5.5% were using the oil recommended by the staff at the nearby Āyurveda pharmacy, and 1.4% selected the oil recommended by the doctor. Homemade oil was used by 62.1% of mothers, whereas 27.6% were using market bought oil. The massage oil was never heated by 86.9%, but 2.8% did always heat the oil before massage (Table 5).

**Table 5:** Selection and usage of oil.

Variable	Category	Count	Percentage
Which oil do you use mostly for head massage	Baby oil manufactured by leading pharmaceutical companies	2	1.4
	Medicated Ayurvedic oil	18	12.4
	Virgin/normal coconut oil	110	75.9
Which oil do you use mostly for body massage	Baby oil manufactured by leading pharmaceutical companies	5	3.4
	Medicated Ayurvedic oil	24	16.6
	Virgin/normal coconut oil	98	67.6
What is the reason for choosing this oil	By traditional knowledge	120	82.8
	Recommended by the doctor	2	1.4
	Recommended by the staff at the nearby Āyurveda pharmacy	8	5.5
Where do you get the massage oil from	Prepare at home on your own	90	62.2
	Purchase from market	40	27.6
Do you preheat the oil before massage	Always	4	2.8
	Never	126	86.9
For pre-heating, which of the following methods do you practice	Directly heat the container over fire	3	2.1
	Double boiling	1	0.7

### 4.6 Assessing relation between education and knowledge

**Figure 1:**
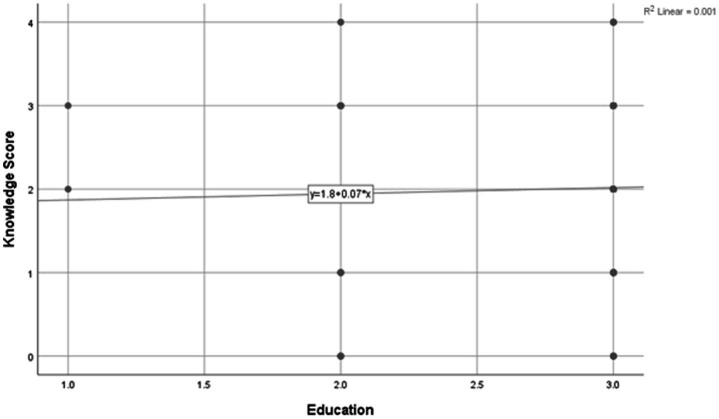
Scattered plot showing the association between education level and knowledge score.

Spearman’s rank-order correlation was conducted to examine the relationship between the educational status of mothers and their knowledge score regarding traditional infant oil massage. The analysis revealed a very weak positive correlation between education and knowledge score (Spearman’s *ρ* = 0.025). However, this association was not statistically significant (*p* = 0.767) at the 5% level of significance (Table 6) (see Figure 1).

**Table 6:** Correlation between education status of mothers and knowledge score.

Correlations				
			Education Status of mothers	Knowledge score
Spearman’s rho	Education	Correlation coefficient	1.000	0.025
		Sig. (two-tailed)		0.767
		*N*	145	145
	Knowledge score	Correlation coefficient	0.025	1.000
		Sig. (two-tailed)	0.767	
		*N*	145	145

The scatter plot illustrates the relationship between educational level (*x*-axis) and knowledge score regarding traditional infant oil massage (*y*-axis). Each point represents an individual participant, while the fitted linear trend line summarizes the overall direction of association.

### 4.7 Compare knowledge vs. practice

**Figure 2:**
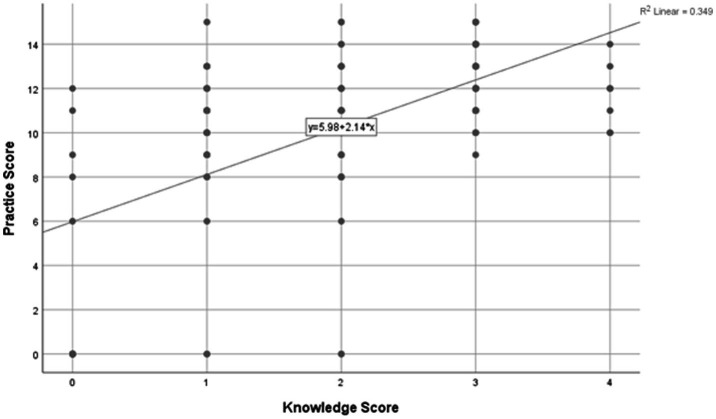
Scattered plot illustrating the relationship between knowledge score (*x*-axis) and practice score (*y*-axis).

The analysis revealed a moderate positive correlation between knowledge and practice scores (Spearman’s *ρ* = 0.524), which was statistically highly significant (*p* < 0.001). This finding indicates that higher levels of knowledge regarding traditional infant oil massage are associated with better and more appropriate practice (Table 7) (see Figure 2).

**Table 7:** Correlation between the knowledge score and practice score.

Correlations				
			Knowledge score	Practice score
Spearman’s rho	Knowledge score	Correlation coefficient	1.000	0.524^**^
		Sig. (two-tailed)		0.000
		*N*	145	145
	Practice score	Correlation coefficient	0.524^**^	1.000
		Sig. (two-tailed)	0.000	
		*N*	145	145

^**^Correlation is significant at the 0.01 level (two-tailed).

The scatter plot depicts the relationship between knowledge score (*x*-axis) and practice score (*y*-axis) related to traditional infant oil massage among the study participants. Each data point represents an individual respondent, while the fitted linear regression line summarizes the overall trend.

The regression equation (*y* = 5.98 + 2.14*x*) indicates that for every one-unit increase in knowledge score, the practice score increases by approximately 2.14 units, reflecting a substantial positive association between knowledge and practice. The coefficient of determination (*R*^2^ = 0.349) suggests that nearly 34.9% of the variability in practice scores can be explained by differences in knowledge levels.

### 4.8 Compare knowledge score between social economical status groups

The Kruskal–Wallis test results indicated that the distribution of knowledge scores did not differ significantly across socio-economic status categories (*p* = 0.514), which is well above the chosen level of significance (*α* = 0.05). Therefore, the distribution of the knowledge score is the same across categories of socio-economic status. This finding suggests that maternal knowledge regarding traditional infant oil massage is uniform across different socio-economic groups. Knowledge related to this practice appears to be independent of economic stratification, likely reflecting its deep cultural embedding and intergenerational transmission within the community rather than access to economic resources or social privilege.

### 4.9 Practice score and socio-economic status

Similarly, the Kruskal–Wallis test showed no statistically significant difference in practice scores across socio-economic status categories (*p* = 0.077). Although this value is closer to the threshold of significance, it still exceeds *α* = 0.05, thus proving the distribution of Practice score is the same across categories of socio-economic status. This result indicates that the actual practice of infant oil massage does not significantly vary with socio-economic status. Mothers from different SES backgrounds appear equally likely to engage in the practice and follow similar procedural patterns, further emphasizing the culturally normative nature of infant oil massage in the study setting.

## 5 Discussion

Knowledge of prevalent complementary practices pertaining pediatric healthcare is an add-on therapy to prevent various ill-health outcomes. This study on traditional child healthcare practices such as oil massage reveals several important details associated with this custom.

### 5.1 Status of infant oil massage practice

It was found that the majority (89.6%) of subjects were either currently giving oil massage to their infants or once practiced it. The observations vividly point out the widespread practice of traditional pediatric oil massage in the study setting. This supports the cultural acceptance of this tradition as we see this similarity in previously reported studies too (4). Recurrent respiratory illness was found to be the main reason for the discontinuation of the practice. This may be due to wrong selection of the oil, mode of application, and body constitution which may have cause respiratory distress in infants (12, 13). Perhaps the cautious practice of daily abhyaṅga might prevent such unpleasant situations. This gap may provide opportunities for Āyurveda physicians to educate the public regarding healthy lifestyle practices as propounded by Āyurveda.

### 5.2 Knowledge of infant oil massage practice

In the course of this study, the finding reveals that 94.5% of the study subjects did not know that abhyaṅga is said in Āyurveda. Many mothers said that they were aware of the conditions to avoid oil massage and accounted only for fever and respiratory illnesses but unaware of indigestion or kaphaja rogas. A large proportion (73.1%) of mothers stated that traditional infant oil massage was beneficial. Previously done studies shown that the knowledge about infant massage among mothers is often rooted in tradition rather than a complete understanding of its scientific benefits and contraindication (14).

### 5.3 Mothers’ attitude toward traditional infant oil massage practice

Almost all mothers commenced the practice as a part of the tradition. Only four did seek the opinion from Āyurveda doctors (via WhatsApp). Therefore, the practice may not be particularly due to inclination toward a healthy lifestyle but owing to the social construct and this is evident form the prior similar studies (15). That could be the reason for this attitude of not seeking proper medical advice from an Āyurvedic medical professional. According to Āyurveda literature, one should pay attention to prakṛti, sātmya, ṛtu, doṣa, and deśa before commencing daily abhyaṅga in healthy person (16). However, a greater number of mothers seem to exhibit a supportive attitude toward traditional infant oil massage practice.

### 5.4 Existing practices of infant oil massage

#### 5.4.1 Commencement of practice

The practice was commenced within 2 weeks after birth by 45.5% mothers, in the third or fourth week by 24.1% and after a month by 20% mothers. Oil massage is strongly recommended by the Indian Academy of Pediatrics with level II evidence, as a skin care practice in neonates (aged 28 days or below) and infants (aged below 1 year) (7). Āyurveda classics recommend jātamātra (neonate) to be anointed with balā taila followed by bath in the context of essential newborn care (jātamātra paricharya) to get rid of the prasūti kleśa (fatigue after birth) (17). The present study observed that the time of commencement of the practice varies in the study setting.

#### 5.4.2 Person providing oil massage

Mother or close relatives (grandmother) provided massage in 82.8% infants. The mother–baby attachment measured by the Maternal Attachment Inventory was found to be increased following mother-given infant massage as evident from previous studies (18).

In addition to sensual contact, it results in an interaction, communication, and emotional bonding between the two which is very important in the early days of life as it improves the baby’s mental-motor development, increases sleep duration, decreases crying, and also enhances response toward mother (19). Hence, the touch in initial days is a baby’s perception of new environment; it is of great significance that who performs the pediatric massage.

#### 5.4.3 Time and duration of oil massage

A very large proportion of the mothers (87.6%) said that they opted the morning timings for the procedure. Āyurveda advocates abhyaṅga (oil massage) as an everyday practice to all the healthy or can be practiced once in 2 days or once in 3 days (20). As abhyaṅga in dinacaryā (daily regimen) is to preserve the health by not disturbing the doṣas, convenient adjustments in terms of its frequency such as in the study setting is expected to be of least harm. The duration of each massage session was not more than 10 min in majority (59.3%) infants. The estimated time taken for deep absorption of the massage medium up to majjā dhātu level is 900 mātrā kāla (16). According to Ārogyacintāmaṇi, an Āyurvedic vernacular text in Malayalam, the time duration for oil anointing in newborn is half nāzhika (approximately 12 min) as explained in jātamātra paricharya (21), but there exist no strict regulations in relation to the duration of daily abhyaṅga in svasthabāla. In addition, the target group here is very vulnerable and it is not easy to keep them calm for too long. Therefore, convenient duration as reported by mothers seems to be not an inappropriate choice.

#### 5.4.4 Procedure

Most of the infants were given abhyaṅga on both head and body including earlobes and feet. According to Āyurveda, it should be more focused on scalp, ears, and feet because head is the master organ and ear lobes and plantar surfaces are said to have numerous nerve endings (20). Pādābhyaṅga is beneficial for eyesight as it stimulates two siras in the plantar surface that are leading to eyes (22). Massage improves circulation and relaxes one as it depresses the sympathetic nervous system, stabilizes the norepinephrine and cortisol levels, and reduces stress by activating parasympathetic nervous system (23, 24). Since almost everyone responded as giving both head and body abhyaṅga inclusive of ears and feet, the observation goes on par with what is said in Āyurveda.

The procedure involved stretching of upper and lower limbs or pressing of specific bodily areas such as the forehead and nose in a few infants, and the scientific community may address this to understand the effect of such steps in determining the overall growth and development. Every child was bathed after massage either immediately or after some time in neutral or lukewarm water. Bathing in warm water (40 °C) could induce a hyperthermic reaction in the body and rise in heart rate and peripheral pO_2_ and fall of pCO_2_, resulting in enhanced metabolism and elimination of wastes which imparts a fresh, revived feeling (25). Hot water is non-conducive for hair and eyes if used daily for head, as per Āyurveda (26).

Cold water for head and warm water medicated with leaves of medicinal plants such as *Ixora coccinea* is advised for body (27). Further studies are anticipated to investigate the health benefits of using such drugs to medicate the water used for bath in children. The effect of time lapse between massage and bath could probably be investigated further in infants, if any. In addition, subsequent studies can perhaps suggest a protocol for infant oil massage adaptable to home settings in the state explaining proper practices and massage techniques.

#### 5.4.5 Selection and usage of oil

The use of an array of nine oils for head and fifteen oils for body massage was explored, and it indicates the variations in the selection of oil among the public (Tables 8, 9).

**Table 8:** Oil used for head.

Sl. No.	Oil used for head	Frequency	Percentage (%)
1	*Jīraka-tulasī siddha keraṃ*	1	0.77
2	Coconut oil + sesame oil	1	0.77
3	Coconut oil/sesame oil/olive oil	1	0.77
4	Himalaya baby hair oil	1	0.77
5	Sebamed baby massage oil	1	0.77
6	Sesame oil	5	3.85
7	*Cemparutyādi keram*	12	9.23
8	Virgin coconut oil	13	10.00
9	Normal coconut oil	95	73.07

**Table 9:** Oil used for body.

Sl. No.	Oil used for body	Frequency	Percentage (%)
1	Almond oil	1	0.79
2	Coconut oil/sesame oil/olive oil	1	0.79
3	*Danthapāla* coconut oil	1	0.79
4	*Jīraka -tulasī siddha keraṃ*	1	0.79
5	*Lākṣādi keram*	1	0.79
6	Sebamed baby massage oil	1	0.79
7	Turmeric oil (vernacular: *Manjhal enna*)	1	0.79
8	Carrot coconut oil	2	1.57
9	Himalaya baby massage oil	3	2.36
10	Coconut milk	4	3.15
11	*Nālpāmarādi keram*	5	3.94
12	Sesame oil	6	4.72
13	*Cemparutyādi keram*	7	5.51
14	Virgin coconut oil	15	11.81
15	Normal coconut oil	78	61.41

The most preferred was coconut oil, probably due to its availability and ease of access (28). It is an emollient and reduces transepidermal water loss, thus maintaining skin integrity (29). Four mothers were using coconut milk extracted from coconut kernel for body massage. Ārogyakalpadrumam particularly mentions coconut milk for yaṅga (offering) in children (21). It is śīta, snigdha, atipuṣṭidā, guru, madhura, vṛṣya, and rakta-pitta-hara. Approximately 38% of the weight of coconut milk is constituted by coconut oil. Therefore, the properties and action of coconut oil may be attributed to coconut milk too. Prolonged use of coconut milk can moisturize and soften the skin and reduce the hyperpigmentation, thus help infants to obtain blemish-free, soft and moist skin.

Massage using olive oil and dantapāla coconut oil was in practice too. Topical application of olive oil on human skin can lose the integrity of stratum corneum and thus becomes a threat to skin barrier function (30). Random suggestion from the neighborhood made one mother choose dantapāla coconut oil where dantapāla is uṣṇa and tīkṣṇa and is used predominantly for the management of psoriasis and seborrheic dermatitis for slowing down the scaling and hyperkeratinization and also as an anti-inflammatory agent (31). It is not advised for healthy individual to do daily massage with this medicated oil. So daily infant oil massage with dantapāla coconut oil may not be a great choice for infants who are saumya twak (gentle and soft skin).

Only less than 4% of mothers chose baby oils manufactured by leading pharmaceutical companies. It may be due to the strong traditional grip of this practice in the community. Although it is an Āyurvedic way of lifestyle to do daily abhyaṅga, the popularity of classical Āyurvedic formulations for infant massage seems limited. Future investigations may be conducted to provide explicable insights into the effects of classical Āyurvedic oils used for massage on skin, growth, development, and overall health of infants.

“Turmeric-coconut oil” (vernacular: “Manjhalenna”) and “carrot-coconut oil” prepared by putting fresh turmeric rhizome or carrot slices in coconut oil for weeks as said by the friends and neighbors were found to be in use for the perceived benefit of improved complexion.

Choice of oil seemed highly reliant on traditional wisdom and culturally accepted. Āyurveda recommends the healthy individuals choose an oil that is in accordance with one’s nature (conducive), season, doṣa, and region whereas coconut milk is explicitly indicated for abhyaṅga of healthy children. So, a balance between traditional and contemporary knowledge may be able to impart a more holistic healthcare approach. Thus, an ideal recommendation that serves as a better infant healthcare measure would be an oil that is suitable for the child’s skin and does not deviate from the aforementioned principles of Āyurveda.

### 5.5 Assessing relation between education and knowledge

Education is generally expected to enhance health-related awareness; however, the negligible correlation observed in this study suggests that knowledge about the infant oil massage in this population may not be strongly associated with formal education. This could be attributed to the culturally transmitted nature of the practice, which is predominantly learned through family traditions, elders, and community norms rather than through formal education or academic exposure (4). As a result, mothers with higher educational qualifications did not demonstrate significantly higher knowledge scores compared to those with lower educational levels.

This lack of association highlights the potential importance of targeted health education interventions, rather than assuming that higher educational status alone is linked with better awareness of traditional or Ayurvedic infant care practices (2). Structured guidance from healthcare professionals, particularly trained Ayurvedic practitioners, may help improve knowledge, irrespective of educational background. However, further longitudinal or interventional studies would be required to establish this effect.

### 5.6 Compare knowledge vs. practice

The study identified a moderate positive correlation between knowledge and practice of infant oil massage. This suggests that higher knowledge levels are associated with more favorable practice patterns. While cultural traditions may initiate the practice, the findings indicate that knowledge could be related to variations in how the practice is carried out. Similar observations have been reported in other studies, where a positive relationship between knowledge and infant massage practices was noted (32). However, given the cross-sectional nature of the study, causal inferences cannot be made regarding whether improved knowledge directly leads to better practice. These findings support the hypothesis that knowledge-based interventions may be beneficial, particularly those focusing on authentic Ayurvedic principles, appropriate techniques, and contraindications. Structured educational efforts delivered by qualified Ayurvedic practitioners could be explored in future studies to evaluate their potential role in improving infant care practices within the community (33).

## 6 Conclusion

Although traditional infant oil massage practice is very popular, the knowledge about it seems fragmentary and varied. The practices differ to a great extent too based on traditional knowledge and the influence of family and neighborhood. Scientific intervention can be useful in this scenario. Systematic guidance including proper training to caretakers and imparting knowledge from reliable sources to parents could be beneficial. Appropriate guidance from a registered Āyurvedic healthcare professional to make the public aware of the authentic Āyurvedic wisdom with logic is expected to contribute to infant healthcare. Thus, Āyurveda can contribute to shaping policies and protocols for care of the infant leading to sculpt a healthy life.

## 7 Limitations and future perspectives

While this study provides valuable insights into traditional infant oil massage practices, certain methodological considerations should be acknowledged. The cross-sectional design limits the ability to infer causality; however, it offers relevant baseline evidence on existing knowledge and practices. The study was conducted in a single district (Malappuram), purposively selected due to its high crude birth rate, enabling adequate sample representation and maintaining socio-cultural consistency, thereby strengthening internal validity. Although this may limit direct generalizability, the findings are likely applicable to similar cultural contexts. The inclusion of primiparous mothers improved sample uniformity but may limit broader applicability. Future research should focus on multi-centric, longitudinal, and interventional designs to enhance external validity, establish causal relationships, and support the development of standardized, evidence-based guidelines integrating Ayurvedic principles with contemporary pediatric care.

## Data Availability

The original contributions presented in the study are included in the article/Supplementary material, further inquiries can be directed to the corresponding author.
