# Supplementary material for: Traditional infant oil massage in early life: a cross-sectional study of knowledge and practices among young mothers in Malappuram district, Kerala
Source: Front Med (Lausanne). 2026 May 8;13:1779228. doi: 10.3389/fmed.2026.1779228 (PMC13195002; doi:10.3389/fmed.2026.1779228)
Supplement: Supplementary file 1 [file Table_1.DOCX]

## Result of tool validation

1. **Content validation**

Table 5.1: Content validation score sheet

| Q. No. | Relevance Scoring:  Questions Scoring 3 or 4 (i.e., agreed) =1  Questions Scoring 1 or 2 (i.e., not agreed) =0 | | | | | Content Validation Index (item level)  I-CVI=  Number of experts giving a relevance rating 3 or 4  ÷  Number of experts |
| --- | --- | --- | --- | --- | --- | --- |
|  | Expert 1 | Expert 2 | Expert 3 | Expert 4 | Expert 5 |  |
| 1 | 1 | 1 | 1 | 1 | 1 | 1 |
| 2 | 1 | 1 | 1 | 1 | 1 | 1 |
| 3 | 1 | 1 | 1 | 1 | 1 | 1 |
| 4 | 1 | 1 | 1 | 1 | 1 | 1 |
| 5 | 1 | 1 | 1 | 1 | 1 | 1 |
| 6 | 1 | 1 | 1 | 1 | 1 | 1 |
| 7 | 1 | 1 | 1 | 1 | 1 | 1 |
| 8 | 1 | 1 | 1 | 1 | 1 | 1 |
| 9 | 1 | 1 | 1 | 1 | 1 | 1 |
| 10 | 1 | 1 | 1 | 1 | 1 | 1 |
| 11 | 1 | 1 | 1 | 1 | 1 | 1 |
| 12 | 1 | 1 | 1 | 1 | 1 | 1 |
| 13 | 1 | 1 | 1 | 1 | 1 | 1 |
| 14 | 1 | 1 | 1 | 1 | 1 | 1 |
| 15 | 1 | 1 | 1 | 1 | 1 | 1 |
| 16 | 1 | 1 | 1 | 1 | 1 | 1 |
| 17 | 1 | 1 | 1 | 1 | 1 | 1 |
| 18 | 1 | 1 | 1 | 1 | 1 | 1 |
| 19 | 1 | 1 | 1 | 1 | 1 | 1 |
| 20 | 1 | 1 | 1 | 1 | 1 | 1 |
| 21 | 1 | 1 | 1 | 1 | 1 | 1 |
| 22 | 1 | 1 | 1 | 1 | 1 | 1 |
| 23 | 1 | 1 | 1 | 1 | 1 | 1 |
| 24 | 1 | 1 | 1 | 1 | 1 | 1 |
| 25 | 1 | 1 | 1 | 1 | 1 | 1 |

Content validation is the capability of the selected items to represent the variables of the construct in the measure. Content validity indexing is of two types- Item level Content Validity Index (I-CVI) and Scale level Content Validity Index (S-CVI). The I- CVI of each item was calculated in Table 5.1. Neither of the questions were dropped as the I-CVI scores were all 1, which was in acceptable range.

S-CVI = sum of item level content validation index scores ÷ number of items S-CVI = 25 ÷ 25 =1

S-CVI score was calculated as 1, which is acceptable.

Out of the 25 questions in the final draft, 16 were subjected to reliability and construct validity tests. The remaining questions were removed from the tests due to their singular matrix.

For the statistical analyses SPSS and SmartPLS-SEM softwares were used for EFA and CFA respectively.

## Exploratory Factor Analysis (EFA)

Table 5.2: Exploratory factor analysis (EFA)

| **Variable** | **Items** | **KMO & Bartlett’s Test of Sphericity** | **Factor Loadings** | **Cronbach’s Alpha** |
| --- | --- | --- | --- | --- |
| Knowledge of infant oil massage practice | Q5 | KMO=0.568  Chi-square=63.809 Sig = 0.000 | 0.870 | 0.931 |
|  | Q6 |  | 0.915 |  |
|  | Q7 |  | 0.837 |  |
| Attitude towards  infant oil massage practice | Q10 | KMO = 0.508  Chi-square = 13.596  Sig = 0.002 | 0.870 | 0.943 |
|  | Q11 |  | 0.934 |  |
| Practice of infant oil massage | Q12 | KMO = 0.510  Chi-square = 33.838  Sig = 0.000 | 0.881 | 0.934 |
|  | Q13 |  | 0.861 |  |
|  | Q14 |  | 0.873 |  |
|  | Q15 |  | 0.903 |  |
|  | Q16 |  | 0.869 |  |
|  | Q18 |  | 0.942 |  |
|  | Q19 |  | 0.853 |  |
| Selection & usage of oil | Q20 | KMO = 0.540  Chi-square = 35.016  Sig = 0.000 | 0.939 | 0.957 |
|  | Q21 |  | 0.895 |  |
|  | Q22 |  | 0.897 |  |
|  | Q23 |  | 0.915 |  |

## KMO and Bartlett’s tests

KMO and Bartlett’s tests for sampling adequacy were done for each domain to measure the suitability of data for factor analysis. The KMO value should not be less than 0.5 and significance level for the Bartlett’s test should be less than 0.05 in order to suggest a substantial correlation in the data. Here, the result in each domain shows that the

KMO values are not less than 0.5 and Bartlett’s test significance values are not exceeding 0.05. Hence the values were in acceptable range, it indicates the factor analysis could be useful.

## Factor loadings

Factor loadings represent the correlation between the observed variables (questions or items in the questionnaire) and the underlying latent factors. Factor loadings should be over 0.5 to be acceptable. Here the results show the factor loadings are greater than 0.5 and are close to 1, indicating a strong correlation between observed variables and latent factors.

## Reliability - Internal consistency

Reliability was tested by means of internal consistency. Coefficient of alpha (Cronbach’s alpha) is the estimate of internal consistency in EFA and should be greater than 0.7. The result shows that the values of Cronbach’s alpha in each domain lies in the acceptable range indicative of internal consistency of the tool. Hence statistically proven the questionnaire is reliable.

## Confirmatory Factor Analysis (CFA)

Table 5.3: Confirmatory factor analysis (CFA)

| **Variable** | **Items** | **AVE** | **CR** |
| --- | --- | --- | --- |
| Knowledge of infant oil massage practice | Q5 | 0.5734056 | 0.758 |
|  | Q6 |  |  |
|  | Q7 |  |  |
| Attitude towards infant oil massage practice | Q10 | 0.6444745 | 0.725 |
|  | Q11 |  |  |
| Practice of infant oil massage | Q12 | 0.5539795 | 0.852 |
|  | Q13 |  |  |
|  | Q14 |  |  |
|  | Q15 |  |  |
|  | Q16 |  |  |
|  | Q18 |  |  |
|  | Q19 |  |  |
| Selection & usage of oil | Q20 | 0.564687 | 0.942 |
|  | Q21 |  |  |
|  | Q22 |  |  |
|  | Q23 |  |  |

Confirmatory Factor Analysis (CFA) is a statistical measure that verifies the factor structure of a set of observed variables.

## Reliability - Internal consistency

Composite Reliability (CR) measures the internal consistency via CFA in SEM analysis. Composite Reliability value must exceed 0.7 in each construct and the result shown as the CR estimates were not less than the desired level.

## Construct Validity - Convergent type

Factor loadings should be greater than 0.5 and here the result shown as all the loadings were at the acceptable level, contributing to the convergent type of construct validity. Average Variance Extracted (AVE) estimate for each of the construct must be over 0.5 and the result shown as the AVE values were in the desired level, indicative of convergence.

Thus established the convergent type of construct validity.

## Construct Validity - Discriminant type

Table 5.4: Discriminant validity

|  | Attitude | Knowledge | Practice | Selection and Usage |
| --- | --- | --- | --- | --- |
| Attitude | **0.734** |  |  |  |
| Knowledge | 0.449 | **0.787** |  |  |
| Practice | -0.17 | -0.131 | **0.783** |  |
| Selection and Usage | -0.592 | -0.552 | 0.093 | **0.791** |

Discriminant validity guarantees that the constructs (or latent variables) are distinct from each other and are measuring different concepts in a study.

According to Fornell and Larcker Criterion, the square root of AVE estimate by a construct should be higher than the correlation with other constructs to establish discriminant validity. The table shows the discriminant validity assessed through Fornell and Larcker Criterion in this study.

## Measurement Model


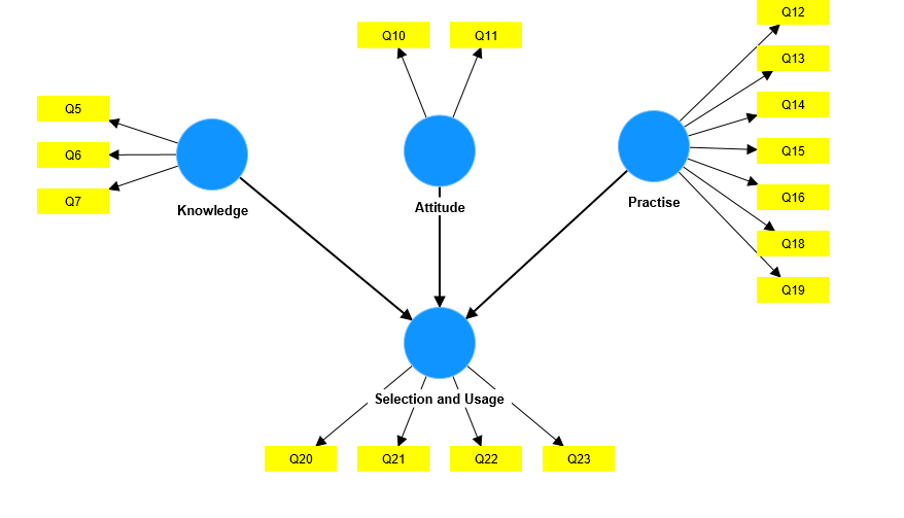


Figure 5.1: Measurement Model Diagram

Measurement model depicts the relationship between a latent variable and its indicators. The measurement model obtained through SEM analysis in the present study is shown in figure 5.1

## Goodness of Model Fit

Table 5.5: Summary of Fit Indices of the Model

| **Goodness of fit index** | **Values** |
| --- | --- |
| SRMR | 0.078 |
| d_ULS | 3.918 |
| d_G | 3.004 |
| Chi-square | 168.673 |
| NFI | 0.876 |

Overall model fit is essential in SEM evaluation. Model fit index determines whether a set of observed values matches those expected under the applicable model.

Table 5.5 shows the Model fit was determined by the indices SRMR, d_ULS, d_G, Chi- square and NFI. The recommended estimates of SRMR should be lower than 0.09 and that of NFI should be closer to 1 for a good model fit. The results show that the model has a good fit.

## Structural Model


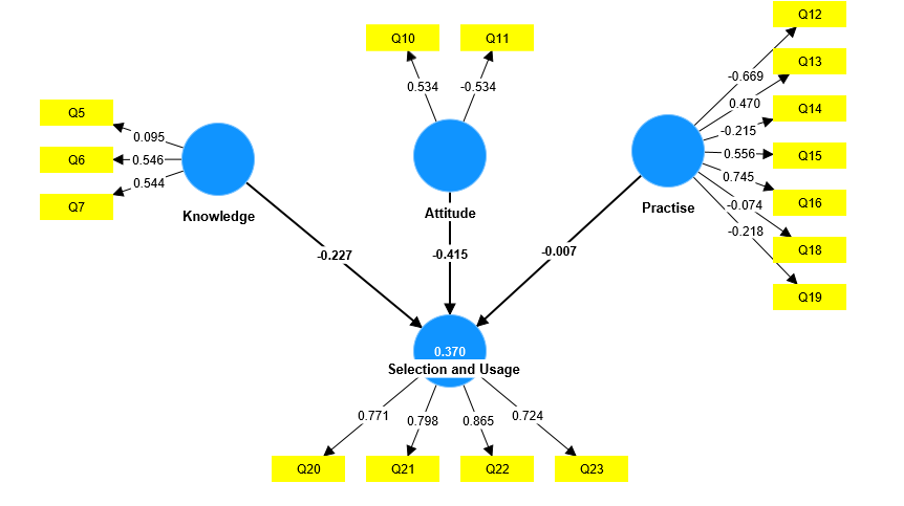


Figure 5.2: Results of Model – SEM

Structural model depicts the relationship between the various constructs in a model. Also specifies how latent variables directly or indirectly affect other latent variables in the model. Figure 5.2 shows the structural model obtained through SEM analysis in the study.
