# Supplementary material for: Traditional infant oil massage in early life: a cross-sectional study of knowledge and practices among young mothers in Malappuram district, Kerala
Source: Front Med (Lausanne). 2026 May 8;13:1779228. doi: 10.3389/fmed.2026.1779228 (PMC13195002; doi:10.3389/fmed.2026.1779228)
Supplement: Supplementary file 2 [file Table_2.DOCX]

**Questionnaire**

“Knowledge and Practices of Traditional Infant Oil Massage in Kerala – A Cross- sectional Survey Study among Young Mothers in Malappuram District”

Subject No.: Date: ............

Health block:

Sub-center:

Details of Mother

1. Name & Age:
2. Address & Mobile number:
3. Religion: Hindu/Christian/Muslim
4. Marital status: married/widow/divorced
5. Education: basic/10th /+2 /university/none
6. Occupation: private sector/public sector/self-employed/housewife
7. Socio-economic status: poor/middle/high

Details of the child

1. Name:
2. Date of birth:
3. Age: ….......months .........weeks days
4. Gender: male/female
5. Birth weight: ….......kg. g
6. Mode of delivery: Vaginal/Abdominal
7. Place of delivery: private hospital/government hospital/private medical college/government medical college/house
8. Duration of pregnancy: weeks

DOMAIN 1 – STATUS OF INFANT OIL MASSAGE PRACTICE

1. Do you practice oil massage for the infant currently?
   1. Yes (please skip to question no.5)
   2. No
   3. Prefer not to say
2. Have you ever given oil massage for the infant?
   1. Never given
   2. Given, but discontinued (please skip to question no.4)
   3. Prefer not to say (please skip to question no.5)
3. What is the reason for not practicing infant oil massage ever?
   1. As advised by registered medical professional
   2. No skilled person available
   3. No specific reason/own choice
4. What was the reason for discontinuing the practice of oil massage?
   1. As advised by the doctor due to recurrent illnesses (fever/respiratory/skin)
   2. No skilled person available
   3. Own choice

DOMAIN 2- KNOWLEDGE OF INFANT OIL MASSAGE PRACTICE

1. Do you know that there is authentic reference for oil massage in Ayurveda?
   1. Yes
   2. No
   3. Prefer not to answer
2. Are you aware of the conditions in which you should avoid giving oil massage?
   1. Yes
   2. No
   3. Uncertain/ don’t know
3. What is your opinion about traditional infant oil massage practice?
   1. Beneficial
   2. Not beneficial
   3. No opinion/ don’t know/ prefer not say

DOMAIN 3 - ATTITUDE TOWARDS INFANT OIL MASSAGE PRACTICE

1. Why did you start giving massage for the baby?
   1. As a traditional practice
   2. As advised by the doctor
   3. As told by the staff at the nearest Ayurveda pharmacy
2. Did you ever seek the opinion of any registered Āyurvedic practitioner before starting the massage practice?
   1. Yes
   2. No
   3. Prefer not to answer
3. Are you planning to continue/restart this practice of oil massage to your baby?
   1. Yes
   2. No
   3. Don’t know/ uncertain
4. Do you recommend infant oil massage to others?
   1. Yes
   2. No
   3. No opinion

DOMAIN 4 - PRACTICE OF INFANT OIL MASSAGE

1. When did you start giving massage to the baby?
   1. Within 2 weeks after birth
   2. After 2 weeks but within 30 days from birth
   3. One month after birth
2. Who gives the massage to your baby generally?
   1. Mother/close relatives
   2. Hired care giver at home
   3. Skilled professional during institutional puerperal care
3. How often is oil massage given to your baby?
   1. Daily
   2. Alternate days
   3. Less than 3 times a week
4. At what time do you oil massage the baby regularly?
   1. Morning
   2. Evening
   3. Depending upon the baby's sleep
5. How long does a massage session last?
   1. Less than 10 minutes
   2. 10 –20 minutes
   3. More than 20 minutes
6. Which part do you oil massage?
   1. Both head and body
   2. Head only
   3. Body only
7. Do you stretch/press any specific areas of the baby's body during massage?
   1. Always
   2. Sometimes
   3. Never
8. What follows the oil massage?
   1. Will retain the oil for a fixed time and later bathe or wipe
   2. Will bathe the baby immediately
   3. Will wipe the baby with a cloth immediately DOMAIN 5 - SELECTION & USAGE OF OIL
9. Which oil do you use mostly for head massage?
   1. Virgin /normal coconut oil
   2. Medicated Āyurvedic oil
   3. Baby oil manufactured by leading pharmaceutical companies
10. Which oil do you use mostly for body massage?
11. Virgin /normal coconut oil
12. Medicated Āyurvedic oil
13. Baby oil manufactured by leading pharmaceutical companies
14. What is the reason for choosing this oil?
    1. Recommended by the doctor
    2. By traditional knowledge
    3. Recommended by the staff at the nearby Ayurveda pharmacy
15. Where do you get the massage oil from?
    1. Prepare at home on your own
    2. Prepare at home according to the instructions of an Ayurveda doctor
    3. Purchase from market
16. Do you pre-heat the oil before massaging?
    1. Always
    2. Sometimes
    3. Never
17. For pre-heating, which of the following methods do you practice?
    1. Directly heat the container over fire
    2. Pour oil into a heated vessel
    3. Double boiling
